# Glutaminase inhibitor CB-839 synergizes with carfilzomib in resistant multiple myeloma cells

**DOI:** 10.18632/oncotarget.16262

**Published:** 2017-03-16

**Authors:** Ravyn M. Thompson, Dominik Dytfeld, Leticia Reyes, Reeder M. Robinson, Brittany Smith, Yefim Manevich, Andrzej Jakubowiak, Mieczyslaw Komarnicki, Anna Przybylowicz-Chalecka, Tomasz Szczepaniak, Amit K. Mitra, Brian G. Van Ness, Magdalena Luczak, Nathan G. Dolloff

**Affiliations:** ^1^ Department of Cell and Molecular Pharmacology & Experimental Therapeutics, Medical University of South Carolina, Charleston, SC, USA; ^2^ Karol Marcinkowski University of Medical Sciences, Poznan, Poland; ^3^ University of Chicago, Chicago, IL, USA; ^4^ Institute of Bioorganic Chemistry, Polish Academy of Sciences, Poznan, Poland; ^5^ University of Minnesota, Minneapolis, MN, USA

**Keywords:** multiple myeloma, proteasome inhibitor, glutaminase, carfilzomib

## Abstract

Curative responses in the treatment of multiple myeloma (MM) are limited by the emergence of therapeutic resistance. To address this problem, we set out to identify druggable mechanisms that convey resistance to proteasome inhibitors (PIs; e.g., bortezomib), which are cornerstone agents in the treatment of MM. In isogenic pairs of PI sensitive and resistant cells, we observed stark differences in cellular bioenergetics between the divergent phenotypes. PI resistant cells exhibited increased mitochondrial respiration driven by glutamine as the principle fuel source. To target glutamine-induced respiration in PI resistant cells, we utilized the glutaminase-1 inhibitor, CB-839. CB-839 inhibited mitochondrial respiration and was more cytotoxic in PI resistant cells as a single agent. Furthermore, we found that CB-839 synergistically enhanced the activity of multiple PIs with the most dramatic synergy being observed with carfilzomib (Crflz), which was confirmed in a panel of genetically diverse PI sensitive and resistant MM cells. Mechanistically, CB-839 enhanced Crflz-induced ER stress and apoptosis, characterized by a robust induction of ATF4 and CHOP and the activation of caspases. Our findings suggest that the acquisition of PI resistance involves adaptations in cellular bioenergetics, supporting the combination of CB-839 with Crflz for the treatment of refractory MM.

## INTRODUCTION

Multiple Myeloma (MM) is the second most common hematological malignancy, accounting for approximately 25,000 new cases and 12,000 deaths per year in the United States, with a 5-year median survival rate of 50% [1]. Treatment advances, particularly the development of targeted therapies like proteasome inhibitors (PIs) and immunomodulatory agents (IMiDs), have dramatically improved treatment response rates and overall patient survival. Despite these developments, nearly all patients eventually progress to a stage of treatment resistance. The refractory setting therefore remains a primary need in MM, and strategies that target molecular mechanisms of resistance to enhance/restore the activity of standard of care drugs are needed for this patient population.

PIs are cornerstone agents in the treatment of MM. They elicit their anti-MM effects through binding to the *PSMb5* subunit of the 26S proteasome and inhibiting the chymotrypsin-like protease activity of the complex. This results in the disruption of normal protein homeostasis and the concomitant induction of cellular proteotoxic stress, thus, proving to be a particularly effective strategy against MM plasma cells, which are naturally designed to mass-produce large multimeric immunoglobulin proteins. It is widely accepted, however, that a variety of molecular mechanisms confer resistance to the cytotoxic effects of PIs. For example, *PSMB5* mutations have been shown to reduce binding of bortezomib (Btz) and carfilzomib (Crflz) to the proteasome in MM and non-MM cell models of PI resistance [2–6], alterations in redox homeostasis have been implicated in protecting MM cells from PI-induced oxidative damage and cell death [6–8], and adaptations involving the cellular protein folding machinery and energy regulation have been implicated in the PI resistance phenotype [6, 9].

Multiple myeloma, like other cancer types, relies on glutaminolysis as a major source of fuel and macromolecular intermediates required for growth and proliferation [10–12]. Recently, it has been demonstrated that MM cells lack expression of glutamine synthetase and display an increased expression of glutaminase 1 (GLS1), suggesting that these cells rely exclusively on extracellular glutamine for cellular energy [13]. GLS catalyzes the conversion of glutamine to glutamate, which supports redox balance through glutathione biosynthesis, and serves as a major substrate for the mitochondrial tricarboxylic acid (TCA) cycle [14–16]. Targeting GLS function using small molecules or gene knockdown approaches has shown promising preclinical anti-cancer activity in breast cancer [17], liver cancer [18], non-small cell lung cancer [19], and B cell lymphoma [18, 20]. The GLS1 selective inhibitor, CB-839, is currently under evaluation for treatment of hematological malignancies and solid tumors, and early indications from a phase 1 study in MM suggests that CB-839 is well tolerated as a monotherapy [21–23]. Preclinical studies have identified synergistic drug combinations with CB-839 in ovarian cancer [24], pancreatic cancer [25], and triple-negative breast cancer [26]. However, for MM, the most effective combinations of CB-839 and standard of care agents have not been determined, thus impeding the rational design of phase 2 combination studies.

In this study, we identified bioenergetic changes in PI resistant MM cells. These adaptations are characterized by an increased reliance on mitochondrial respiration and utilization of glutamine. Targeting glutamine metabolism with CB-839 synergistically enhanced the cytotoxic effects of PIs with the most robust synergy being observed with the second-generation PI Crflz. At the molecular level, we found that CB-839 significantly enhanced Crflz-induced ER stress and apoptotic cell death, providing mechanistic basis for the efficacy of the combination. Our findings suggest that adaptations in cellular bioenergetics are key events in the acquisition of PI resistance, and can be exploited therapeutically by targeting glutamine metabolism. Furthermore, this work identifies the combination of CB-839 and Crflz as a promising combination strategy for refractory MM patients.

## RESULTS

### PI resistant MM cells show heightened levels of mitochondrial respiration

**Figure 1:**
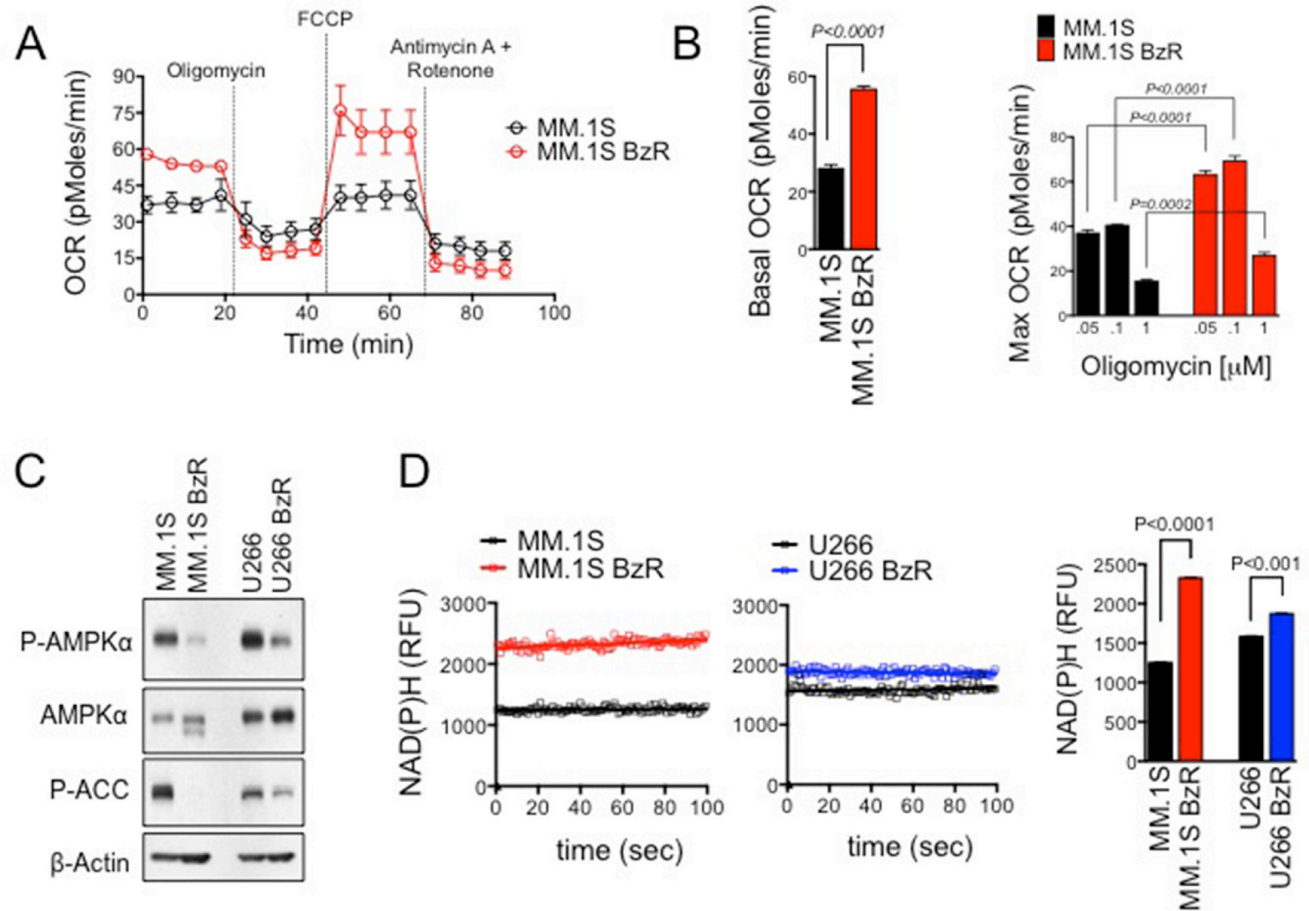
PI resistant cells show increased rates of mitochondrial respiration for energy production (**A**) MM.1S and MM.1S BzR cell lines were plated in XF running buffer and exposed to the Seahorse XF Cell Mito Stress Test, consisting of automated treatment with oligomycin, FCCP, and the combination of antimycin A and rotenone at the indicated times. The oxygen consumption rate (OCR) was measured over time using the XF Extracellular Flux Analyzer. A representative full time course of the XF Cell Mito Stress Test is shown. (**B**) Basal OCR (left) and maximum OCR (right) are shown for 4 independent experiments using cells of various passage number that were analyzed as described in (A). For the determination of maximum OCR, three different concentrations of oligomycin (0.05, 0.1, or 1 μM) were used. Note that regardless of the oligomycin concentration used, the MM.1S BzR cells showed significantly higher max OCR. *P*-values and statistical significance were determined using student's *t-test*. (**C**) Western blots are shown for untreated MM.1S, MM.1S BzR, U266, and U266 BzR cells [P-AMPKα: phospho-AMP kinase alpha (Thr172); ACC: acetyl-CoA carboxylase (Ser79)]. (**D**) Inherent cellular fluorescence of NAD(P)H was measured in untreated cells. Kinetic data from representative experiments are shown (left). The average NAD(P)H ± S.E. levels over a 100 second timeframe are shown (right). A student's *t-test* was used to evaluate statistical significance.

To uncover potential differences in cellular bioenergetics between PI resistant and sensitive MM cells, we analyzed glycolytic and mitochondrial respiration function in paired isogenic cell lines. These isogenic cell models were established by exposing parental human MM cells to progressively higher concentrations of Btz for a period of at least 6 months. Resistant (BzR) cells show significant resistance to Btz as well as cross-resistance to other PIs (carfilzomib, ixazomib, and oprozomib; Supplementary Figure 1). BzR cells were negative for *PSMB5* gene mutations that have been reported by others in PI resistant cell models (Supplementary Figure 2), [4, 29] suggesting that resistance is the result of cellular adaptations that allow cells to evade the cytotoxic effects of PIs rather than mutational events that interfere with PI binding and inhibition of the proteasome. Using the XF-96 extracellular flux analyzer, we measured oxygen consumption rate (OCR) and extracellular acidification rate (ECAR) to assess rates of mitochondrial respiration and glycolysis, respectively. We found that PI resistant cells had a significantly higher basal OCR and mitochondrial respiratory capacity (Figure 1A–1B, Supplementary Figure 3A) compared to sensitive parental cells. We did not detect any significant differences in ECAR between sensitive and resistant cells (Supplementary Figure 3B), suggesting that sensitive and resistant cell lines have similar rates of glycolysis. We observed additional divergence in bioenergetic biomarkers between PI sensitive and resistant cell lines. Under resting/untreated conditions, BzR cells had significantly lower levels of AMP kinase (AMPK) phosphorylation and phosphorylation/activation of the downstream AMPK substrate acetyl-CoA carboxylase (ACC; Figure 1C). AMPK is an energy stress response kinase activated in response to an increase in the cellular AMP:ATP level. Lower levels of AMPK phosphorylation/activation suggest that BzR cells are more energetically efficient and less prone to energy stress, an inference that is consistent with having an increased mitochondrial respiratory capacity, which carries the ability to more efficiently generate cellular fuel in the form of ATP. Furthermore, in BzR cells, we detected increased levels of NAD(P)H, which serve as electron carriers in the mitochondrial process of oxidative phosphorylation (Figure 1D). All together, these findings demonstrate that the PI resistance phenotype is associated with changes in cellular bioenergetics that favor the increased use of mitochondrial respiration for energy production.

### Increased mitochondrial biomass is associated with PI resistance in cell models and in MM patient plasma cells

**Figure 2:**
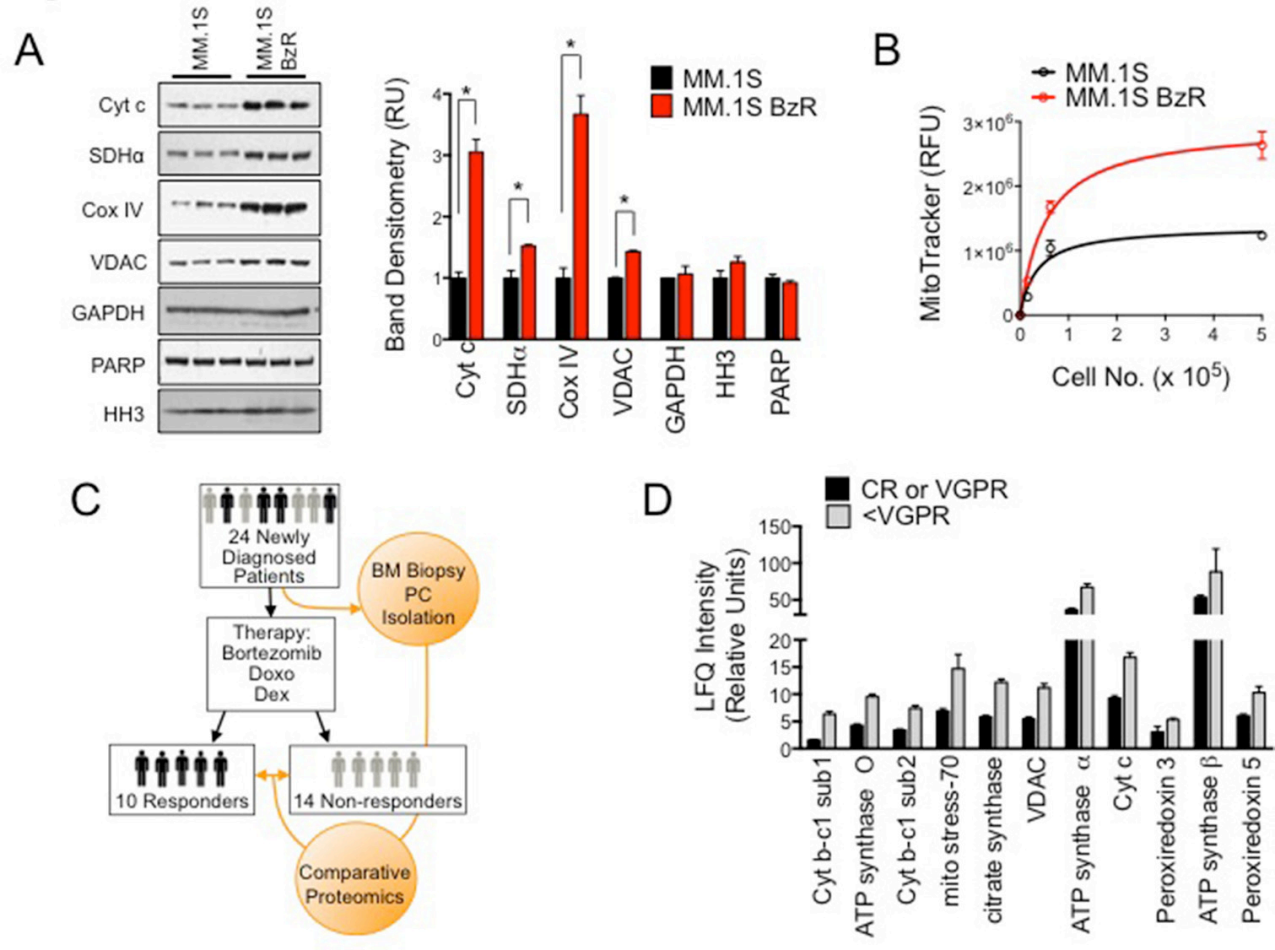
Mitochondrial biomarkers are enriched in PI resistant cell lines and primary plasma cells from MM patients that responded poorly to a Btz-based treatment regimen (**A**) Western blots are shown for the indicated mitochondrial, cytoplasmic, and cytosolic markers (Cyt c: cytochrome C; SDHα: succinate dehydrogenase alpha; Cox IV: cytochrome C oxidase, GAPDH: glyceraldehyde 3-phosphate dehydrogenase, PARP: poly ADP ribose polymerase, HH3: histone H3) (right). Band densitometry from the western blot are shown (left). Statistical significance was determined using a student's *t-test* (*P < 0.05, *N* = 3). (**B**) MitoTracker relative fluorescence was measured in MM.1S and MM.1S BzR cells incubated with 200 nM of MitoTracker Green FM probe. The average relative fluorescent unit (RFU) ± S.E. is shown for a range of cell numbers in triplicate. (**C**) Schematic of clinical data set acquisition. Bone marrow aspirates were collected from newly diagnosed MM patients prior to the induction of PAD therapy (Bortezomib, Doxo: doxorubicin; Dex: dexamethasone). Objective responses were evaluated and patients were categorized as Responders (VGPR or better) or Non-responders (< VGPR). MM plasma cells were purified and subjected to comparative proteomics to identify protein biomarkers that are enriched in MM plasma cells from patients that are less likely to respond favorably to a Btz based treatment regimen. (**D**) Comparative proteomics data from the trial described in (C) are shown. Label-free quantification (LFQ) intensities for the indicated mitochondrial markers are shown, comparing intensities (i.e. expression levels) from patients in the Responders (black bars, *N* = 10) and Non-responders (gray bars, *N* = 14) groups. All differences between groups were determined to be statistically significant (*P* < 0.05) using a students *t-test*.

We hypothesized that PI resistant MM cells might have increased mitochondrial biomass, which would account for their higher mitochondrial respiratory rates. To examine this possibility, we first quantified the expression of a panel of mitochondrial biomarkers and compared levels between PI sensitive and resistant cells. PI resistant MM.1S cells expressed significantly higher levels of several mitochondrial markers, including cytochrome c (Cyt C), succinate dehydrogenase alpha (SDHα), cytochrome C oxidase IV (Cox IV), and voltage dependent anion channel (VDAC), compared to PI sensitive MM.1S cells (Figure 2A). By comparison, both cell types expressed equal amounts of the cytosolic marker glyceraldehyde-phosphate dehydrogenase (GAPDH) and the nuclear markers histone H3 (HH3) and poly ADP ribose polymerase (PARP). We also quantified the amount of mitochondrial specific dye (MitoTracker) staining in PI sensitive versus resistant cells. PI resistant cells showed greater mitochondrial specific fluorescence, providing further evidence of increased mitochondrial biomass in PI resistant cells (Figure 2B). Thus, the adaptation to PI therapy and acquisition of the PI resistant phenotype is accompanied by an increase in mitochondrial biomass, which supports and explains, at least in part, the enhanced mitochondrial respiration rates of PI resistant cells.

We next investigated the link between increased mitochondrial biomass and reduced responsiveness to PI therapy in clinical specimens from MM patients treated with a Btz-based treatment regimen. At diagnosis and prior to the initiation of treatment, MM plasma cells were isolated from patient bone marrow aspirates. All patients in the study received Btz, doxorubicin, and dexamethasone (PAD). Response to the treatment was evaluated according to International Myeloma Working Group Recommendation [28]. Patients were grouped as responders if they achieved at least a VGPR (i.e., CR and VGPR), or non-responders if they achieved a lower response (i.e., PR and PD; study design overview shown in Figure 2C). Comparative proteomics was then conducted on the treatment naïve plasma cells in order to correlate the expression of cellular protein biomarkers with response/resistance to therapy. Out of 2871 proteins identified (false discovery rate, FDR < 1%), 73 showed significant differences between responder and non-responder patients receiving PAD (Supplementary Table 1A–1B). Thirty-seven (37) proteins were downregulated (Supplementary Table 1A) in non-responders, while 36 were upregulated (Supplementary Table 1B). For proteins that were significantly up-regulated or enriched in the non-responder group (defined by a greater than 1.5-fold increased in expression), we found that many of the top hits were mitochondria resident proteins including components of the cytochrome b-c and cytochrome c complexes (UQCRC1, UQCRC2, and CYCS), ATP synthase subunits (ATP5O, ATP5A1, ATP5B), mitochondrial resident proteins involved in regulating mitochondrial redox potential (PRDX3 and PRDX5), enzymes involved in the TCA cycle (CS), and mitochondrial channel proteins (VDAC; Figure 2D). These findings corroborate our data using cell models of PI resistance as they indicate that patients that are less likely to respond to a Btz-based therapeutic regimen have MM plasma cells that are enriched with mitochondrial biomarkers. We conclude from these preclinical and clinical studies that increased mitochondrial respiration and increased mitochondrial biomass is a trait of MM plasma cells with acquired or inherent resistance to PI therapy. They further suggest that a strategy targeting mitochondrial respiration may enhance the activity of PIs.

### Glutamine is the primary fuel source driving cell proliferation and mitochondrial respiration in MM cells

**Figure 3:**
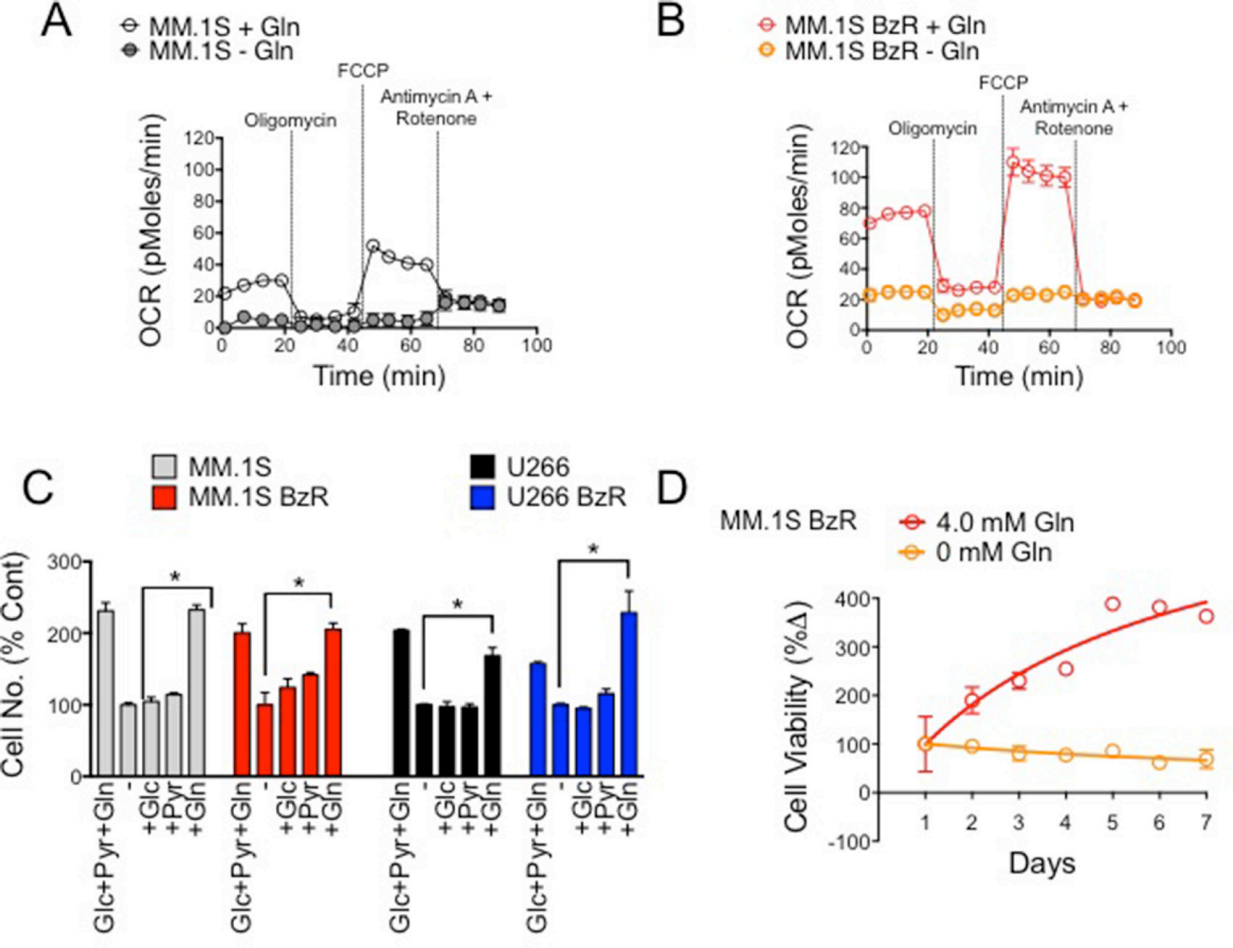
Glutamine is the primary fuel source driving mitochondrial respiration, cell proliferation and survival in PI sensitive and resistant MM cells (**A**) Mitochondrial respiration (OCR) was measured in PI sensitive MM.1S cells in the presence and absence of 4 mM glutamine (Gln). Representative data of 3 independent experiments are shown. (**B**) Mitochondrial respiration (OCR) was measured in PI resistant MM.1S BzR cells in the presence and absence of 4 mM glutamine (Gln). Representative data of 3 independent experiments are shown. (**C**) PI sensitive and resistant MM.1S and U266 cells were incubated in cell media lacking (−) or containing the indicated nutrients (Glc: glucose, 2 g/L; Pyr: pyruvate, 100 mM; Gln: glutamine, 4.0 mM) for 72 hours. Live cell numbers were quantified using a Cellometer (Nexelom) and associated software. Data were normalized to the experimental group lacking nutrients (−). (**D**) MM.1S BzR cells were incubated in cell media with or without 4.0 mM glutamine (Gln) beginning at time 0. Cell viability was measured every 24 hours for 7 days total.

To identify potential molecular strategies for disrupting mitochondrial respiration, we first set out to determine the critical fuel source(s) used by MM cells to drive respiration. We found glutamine to be the principle source of fuel for both PI sensitive and resistant MM cells as removing glutamine from the media was sufficient to almost completely suppress basal mitochondrial respiration and overall mitochondrial capacity (Figure 3A–3B). Furthermore, the presence of glutamine was necessary and sufficient to promote the proliferation of multiple MM cells (Figure 3C). Glucose and sodium pyruvate were dispensable, as their supplementation in the absence of glutamine was unable to induce proliferation above the rates seen in fuel deficient media. By comparison, the reintroduction of glutamine alone was able to restore proliferation to the levels seen in growth media containing all three nutrients. Likewise, the viability of PI resistant MM.1S cells failed to increase over a period of seven days when grown in media with no glutamine (Figure 3D). These results demonstrate that glutamine is the principle fuel source driving mitochondrial respiration and cell proliferation in PI sensitive and resistant MM cells. Furthermore, given that enhanced mitochondrial respiration is a trait associated with the PI resistance phenotype, these findings suggest that a therapeutic approach to specifically disrupt glutamine metabolism may be an effective strategy for targeting PI resistant MM.

### Glutaminase 1 inhibitor, CB-839, blocks mitochondrial respiration and enhances carfilzomib induced cell death in MM cells

**Figure 4:**
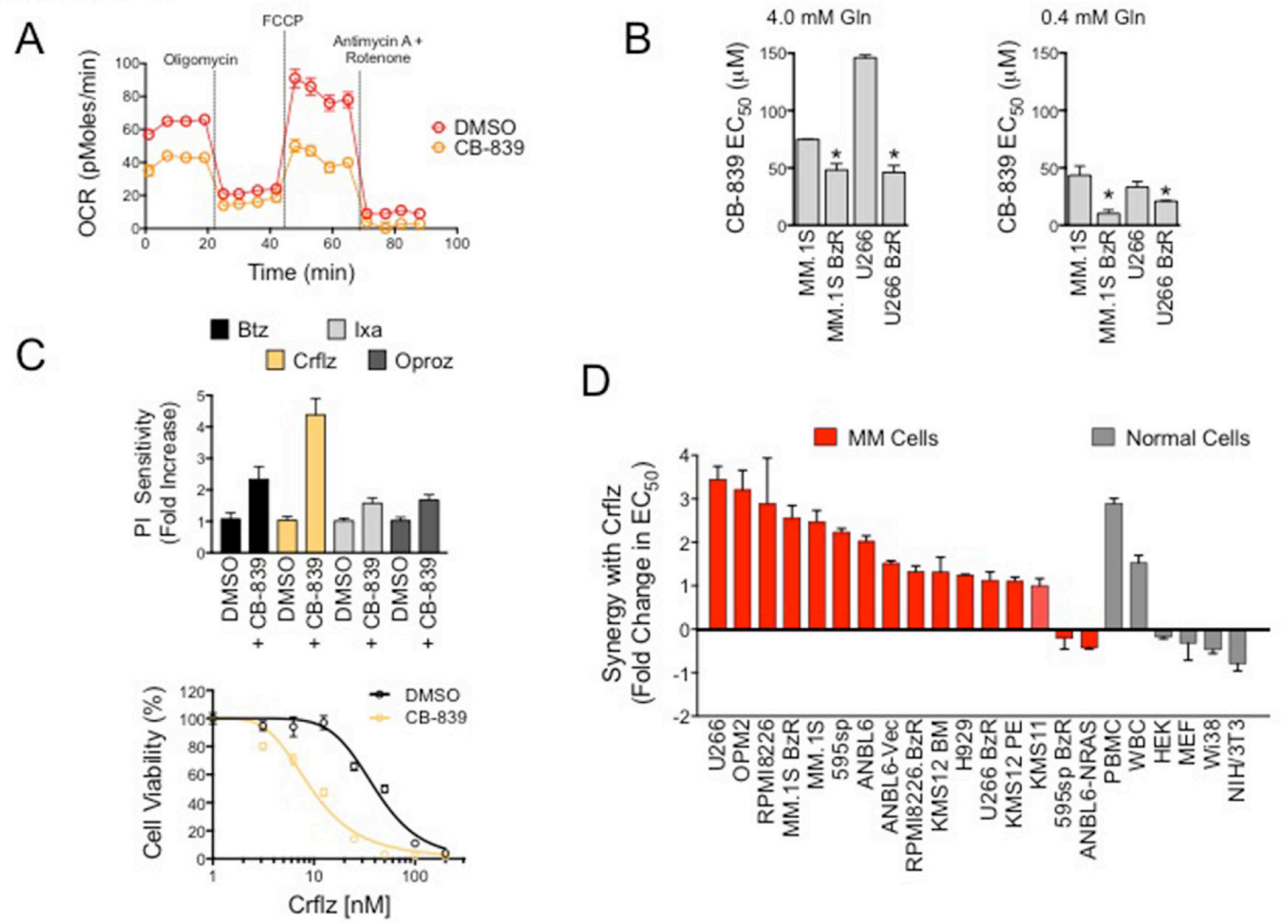
CB-839 inhibits mitochondrial respiration and synergistically enhances PI-induced cytotoxicity in PI resistant and sensitive MM cells (**A**) MM.1S BzR cells were treated with vehicle control (DMSO) or CB-839 (5 μM) for 16 hours. Representative data of mitochondrial respiration rate (OCR) data from 3 independent experiments are shown. (**B**) PI resistant and sensitive MM.1S and U266 cell lines treated with a dose range of CB-839 in media containing 4.0 mM glutamine (left) or 0.4 mM glutamine (right). Cell viability was measured after 72 hours of treatment. CB-839 EC_50_ values for each of the indicated cell lines are shown. Statistical significance was determined by student's *t-test* (*P < 0.05, *N* = 3). (**C**) MM.1S BzR cells were treated with a dose range of 8 concentrations of the indicated PIs (Crflz: carfilzomib and Btz: bortezomib = 0–200 nM; Ixa: ixazomib and Oproz: oprozomib = 0–2 μM) in the presence or absence of CB-839 (5 μM) for 24 hours. Cell viability was then measured and EC_50_ values were extrapolated from each dose response curve. CB-839 at this concentration and duration of treatment had no effect on cell viability by itself, and therefore, any leftward shift in the PI dose response curve was indicative of a superadditive/synergistic drug interaction. Data are represented as relative PI sensitivity (top), where an increase in sensitivity is indicative of a reduction in the absolute EC_50_ of PI. Thus, a 4-fold increase in PI sensitivity indicates that in the presence of CB-839 4 times less PI is required to kill 50% of the cells. A representative dose response curve for Crflz is shown (bottom). (**D**) A panel of PI resistant and sensitive MM cells (in red) and normal human primary cells and fibroblast cell lines (gray) were treated with a dose range of Crflz in addition to DMSO (control) or CB-839 (5 μM) for 24 hours. Synergy between CB-839 and Crflz is represented as the fold change in Crflz EC_50_, where, as in (C), a reduction in the absolute Crflz EC_50_ is indicative of a relative increase in synergy.

Given the increased reliance of PI resistant MM cells on mitochondrial respiration, and the critical role of glutamine for cellular respiration, we reasoned that inhibition of glutamine metabolism was a rational molecular strategy for the treatment of PI resistant MM. To target glutamine metabolism, we used the glutaminase 1 inhibitor, CB-839. We found that treatment with CB-839 significantly repressed OCR in PI resistant cells (Figure 4A). Treatment with CB-839 for 72 hours reduced the viability of both PI sensitive and resistant cells. PI resistant MM.1S BzR and U266 BzR cells were more sensitive to single agent CB-839 with EC_50_ values that were significantly lower than those observed for parental/sensitive MM.1S and U266 cells (Figure 4B). The effects of CB-839 on cell viability were inversely proportional to the concentrations of glutamine in culture. For example, CB-839 showed more potent cytotoxic effects in the presence of 0.4 mM, which is representative of the physiological glutamine levels, [29] compared to 4.0 mM glutamine (Figure 4B). To test for potential synergy between CB-839 and PIs, we then treated PI resistant cells with a fixed concentration of CB-839 (5 μM) and a dose range of four PIs, bortezomib (Btz), carfilzomib (Crflz), ixazomib (Ixaz), and oprozomib (Oproz). CB-839 synergistically enhanced the activity of all PIs (Supplementary Figure 4), but the most dramatic effect was observed when CB-839 was combined with Crflz with doses as low as 156 nM CB-839 (Figure 4C, Supplementary Figure 5). Note that the effect of 5 mM CB-839 alone after 24 hours of treatment was negligible (Supplementary Figure 6), and therefore the leftward shift of the Crflz dose response curve is indicative of a superadditive/synergistic interaction between the two drugs. These findings were validated using a panel of 16 PI resistant as well as sensitive, genetically diverse MM cell lines. The magnitude of synergy between CB-839 and Crflz ranged from 2 to 4 fold (Figure 4D, Supplementary Table 2). In MM.1S BzR cells, for example, co-treatment with CB-839 reduced the EC_50_ for Crflz from 42.3 nM to 10.2 nM, an approximately 4-fold increase in sensitivity. We also evaluated the effects of CB-839 on Crflz sensitivity in a panel of normal human primary cells and cell lines. CB-839 increased the sensitivity of primary normal human peripheral blood mononuclear cells (PBMCs) and lymphocytes (white blood cells; WBCs) while decreasing/protecting fibroblasts cell lines (HEK, MEF, Wi38, and NIH3T3) from the cytotoxic effects of Crflz. Together, these data demonstrate that CB-839 effectively inhibits glutamine metabolism and mitochondrial respiration rates, two critical bioenergetic components of PI resistant MM cells. They further show that CB-839 synergizes with PIs, particularly Crflz, enhancing their cytotoxic activity in both PI sensitive as well as resistant MM cells.

### CB-839 enhances Crflz-induced apoptosis and ER stress

**Figure 5:**
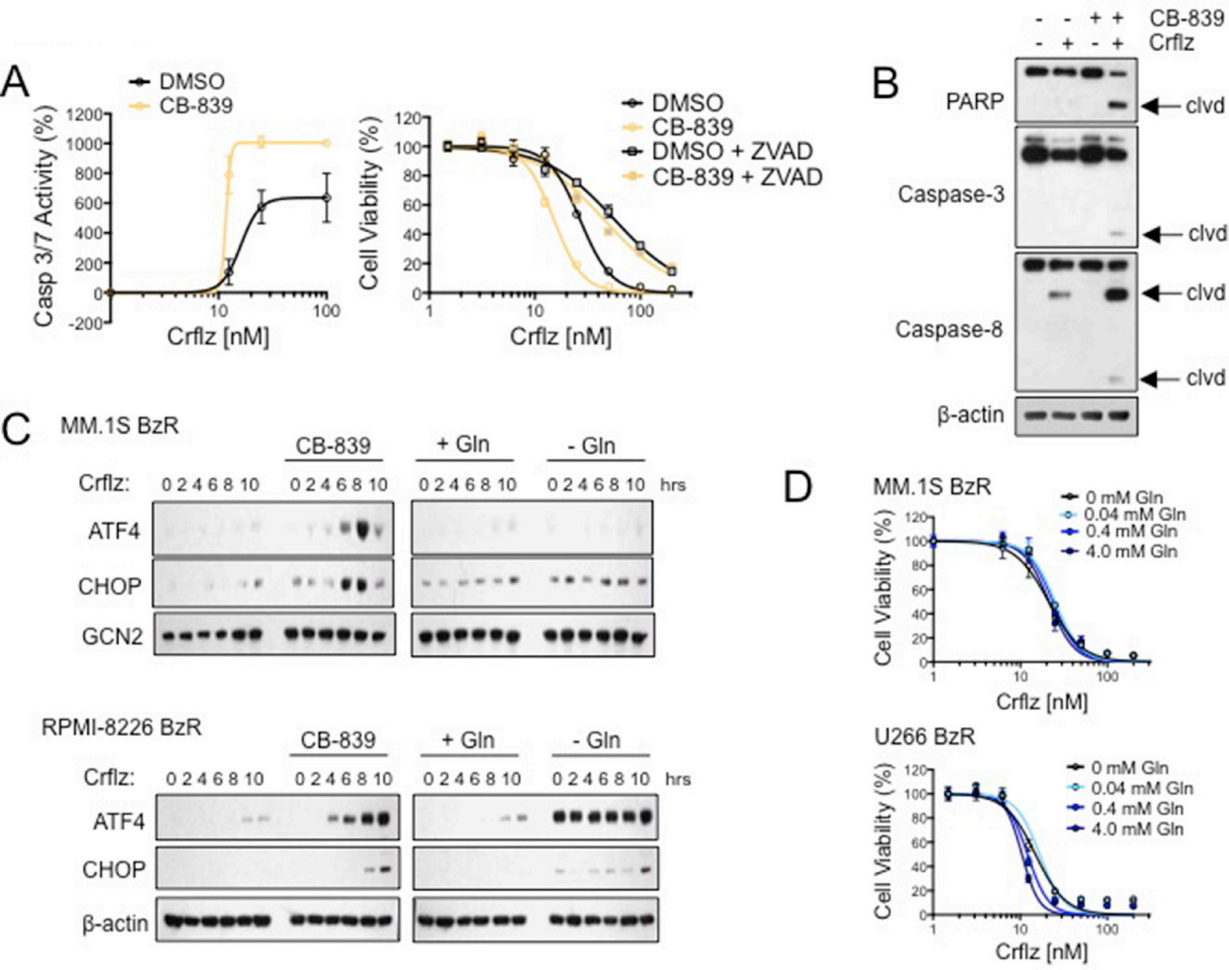
CB-839 synergistically enhances Crflz-induced apoptosis and ER stress (**A**) MM.1S BzR cells were treated with a dose range of Crflz in the presence or absence of CB-839 (5 μM). Caspase 3/7 activity was measured after 16 hours (left) and cell viability was measured after 24 hours (right). The pan caspase inhibitor Z-VAD-FMK (20 μM) was added to evaluate the contribution of caspase activity to the cytotoxic activity of CB-839 and Crflz combinations. (**B**) MM.1S BzR cells were treated with DMSO (control), Crflz (20 nM), CB-839 (5 μM), or a combination of Crflz and CB-839 for 16 hours. Western blots are shown (clvd: indicates the cleaved/active forms of caspases or the degradation product of PARP). (**C**) MM.1S BzR cells (top) and RPMI-8226 BzR cells (bottom) were treated for the indicated time points with Crflz (20 nM) alone or in combination with CB-839 (5 μM). For comparison, similar experiments were conducted in media containing or lacking glutamine (4.0 mM). Western blots are shown. GCN2 and β-actin were used as loading controls. (**D**) MM.1S BzR cells (top) and U266 BzR cells (bottom) were treated with a dose range of Crflz in media containing varying concentrations of glutamine for 24 hours, similar to the design of experiments involving CB-839 and Crflz co-treatment. Cell viability data are shown.

We next set out to characterize the molecular effects underlying the synergistic interaction between CB-839 and Crflz. We first examined the effects of CB-839 on Crflz-induced apoptosis. Using a bioluminescence-based assay that measures the activity of caspase-3/7, two executioner caspases in the apoptotic cascade, we found that CB-839 significantly increased Crflz-induced apoptosis in PI resistant MM.1S BzR cells (Figure 5A). The addition of the pan caspase inhibitor, Z-VAD-FMK, nearly completely ameliorated the cytotoxic effects of the CB-839/Crflz combination, confirming a predominant role of apoptotic cell death in the synergy between CB-839 and Crflz (Figure 5A). Furthermore, we found that CB-839 increased the proteolytic processing/activation of apoptotic mediators caspase-3 and caspase-8, and enhanced the proteolysis of the caspase-3 substrate poly ADP ribose polymerase (PARP; Figure 5B). CB-839 had no effect on the ability of Crflz to inhibit the 26S proteasome, suggesting that the synergy between the two agents was not due to effects on cellular drug transporters such as ABCB1, which has been shown by others to contribute to Crflz resistance. [6, 30] Evidence for this is that we observed nearly identical levels of Crflz-induced inhibition of the 26S chymotrypsin-like (CT-L) activity in MM cells in the presence or absence of CB-839 (Supplementary Figure 7). The synergy between CB-839 and Crflz also appeared to be independent of effects on cellular redox biology. Glutamate, the metabolite of GLS1 activity, is one of the amino acid building blocks used in the synthesis of glutathione (GSH), a critical regulator of cellular redox biology. This led us to hypothesize that CB-839 might reduce GSH and impair redox homeostasis, thus making cells more susceptible to the cytotoxic effects of Crflz. To test this hypothesis we supplemented the cells with excess amounts of GSH in the form of a cell permeable GSH ethyl ester (GSH-EE). Treatment with GSH-EE was unable to reverse the synergy between CB-839 and Crflz (Supplementary Figure 8), demonstrating that the PI-sensitizing effects of CB-839 were independent of effects on GSH and redox.

One of the molecular effects of proteasome inhibition is the accumulation of proteins within the cell, leading to the induction of the unfolded protein response (UPR) and endoplasmic reticulum (ER) stress. MM plasma cells, which are professional secretory cells charged with the mass production of large immunoglobulin proteins, are particularly sensitive to disruptions in cellular protein turnover/homeostasis. We therefore next investigated the effects of CB-839 on Crflz-induced ER stress signaling. In PI resistant cells (MM.1S BzR, RPMI-8226 BzR) we found that Crflz treatment alone induced only a modest time-dependent increase in the ER stress markers ATF4 and CHOP. However, co-treatment with CB-839 substantially increased the magnitude of ER stress pathway induction in both cell lines (Figure 5C). Similar results were obtained using U266 BzR cells (Supplementary Figure 10). The combination of CB-839 and Crflz strongly induced eIF2α phosphorylation along with ATF4 and CHOP up-regulation, we did not detect significant differences in XBP-1 splicing or ATF6 induction (Supplementary Figure 11), suggesting that the PERK-eIF2α-ATF4-CHOP axis is the critical arm of the ER stress response activated by CB-839 and Crflz combinations. ER stress markers were strongly induced by glutamine withdrawal alone in RPMI-8226 BzR cells, however those levels were not potentiated by the addition of Crflz as was observed with CB-839 treatment. Interestingly, we found that the effects of CB-839 were not recapitulated by glutamine deprivation, as glutamine withdrawal from the cell media had no effect on Crflz-induced ER stress pathway signaling (Figure 5C, Supplementary Figure 9–10). Furthermore, glutamine deprivation was unable to enhance the cytotoxic effects of Crflz (Figure 5D), drawing a further distinction between inhibition of glutamine metabolism by glutaminase 1 using CB-839 and the partial or complete removal of glutamine from the cell culture conditions. These results demonstrate that CB-839 synergistically enhances Crflz-induced apoptosis and ER stress signaling, providing a mechanistic explanation for the synergistic cytotoxic effects of this drug combination.

## DISCUSSION

MM remains an incurable disease, emphasizing the need for additional therapies toward the ultimate goal of curability. A rational approach to new drug discovery programs is to develop therapeutics that enhance or restore the activity of FDA approved standard of care agents. Previous approaches for enhancing PIs in multiple myeloma have been attempted with cannabinoids, [31] inhibitors of cellular inhibitor of apoptosis 2 [32], and panobinostat, a pan-deacetylase inhibitor [33]. The strategy we have taken is to use isogenic cell models of PI resistance [34], as PIs are cornerstone MM agents for which resistance invariably develops. Isogenic models offer the opportunity to uncover potentially novel mechanisms that confer resistance as well as provide a platform for cell based drug screening for chemical structures that restore/enhance the activity of PIs in resistant MM cells. In this study we show a major change/adaptation that characterizes PI resistant MM cells is an increased capacity and reliance on mitochondrial respiration for energy production, which appears to be the result of increased mitochondrial biomass. Our clinical data sets further support our cell model data, as MM patients that failed to respond to a Btz-based treatment regimen were found by proteomic profiling to express significantly higher levels of mitochondrial biomarkers compared to responders. Having uncovered this mitochondrial-centric bioenergetics program of PI resistant cells, we set out to target mitochondrial respiration for therapeutic gain. We found that glutamine was the critical fuel source driving mitochondrial respiration in MM cells, making glutamine metabolism a rational target for re-sensitizing cells to PIs. To accomplish this we employed the clinical stage GLS1 specific inhibitor, CB-839. In a phase 1 dose escalation trial, CB-839 was well tolerated as a monotherapy in advanced MM patients [21]. Safety and tolerability were the primary endpoints of this study, but preliminary efficacy as a single agent was limited to long term stable disease, suggesting that CB-839 might be most effective in combination with other anti-MM agents. However, it is not clear which standard of care MM agents are best suited for combination with CB-839. Our work identifies the specific use of CB-839 in combination with PIs. CB-839 exhibited varying degrees of synergy with the four PIs that we tested (i.e., Btz, Crflz, Ixa, Oproz), however the synergy was most potent and markedly superior in combination with Crflz. Therefore, this study has translational implications, suggesting that the combination of CB-839 and Crflz may be highly efficacious in MM patients, including those that are refractory to PIs.

It is not clear how increased mitochondrial respiration provides a selective advantage that confers resistance to PI therapy. However, others have recently reported similar findings using independent MM cell models of PI resistance, suggesting that this is a critical adaptation. For example, Soriano and colleagues showed similar increases in mitochondrial respiration and cellular OCR in Amo-1 MM cells that had been selected for Btz and Crflz resistance [6]. On the surface, our findings seem to conflict with the Warburg hypothesis, which originally described the preferential utilization of glycolysis over mitochondrial respiration by cancer cells [35, 36]. A major distinction is that our study focused specifically on treatment resistant cancer cells. So while it is true that cancer cells exhibit higher rates of glycolysis than normal cells, within tumor cell populations, the cells with the highest respiration rates may be those that give rise to treatment refractory disease. In fact, this observation has been reported in other tumor models of therapy resistance as well, and was deemed a potential “chink in the armor” of therapy resistant cancer. [37] One possible explanation is that this divergent bioenergetics program, which relies more heavily on mitochondrial respiration for the generation of ATP, is more versatile and efficient, providing a survival advantage to MM cells in the face of therapy induced stress. A second hypothesis we would propose is explained by the theory of hormesis, a process of adaptation to stress that has been shown to provide a survival advantage to cells and organisms. [38–41] The hormesis paradigm explains that periods of low-level stress trigger cytoprotective adaptations that protect cells or organisms against further stress. The common low-level stressor in these models was reactive oxygen species (ROS). The vast majority of cellular ROS are generated by the process of oxidative phosphorylation within the mitochondria. As higher rates of respiration in PI resistant MM cells are likely to increase ROS byproducts, it is conceivable that these cells have been forced to adapt to increased basal levels of oxidative stress, and those adaptations provide protection from PI induced cell death.

We made the interesting and perhaps unexpected observation that CB-839 treatment and glutamine deprivation do not phenocopy each other in terms of their impact on Crflz-induced cell death and activation of the ER stress pathway. While previous studies investigating the effects of CB-839 have demonstrated similar activity between CB-839 treatment and glutamine deprivation in cancer cells [13, 26, 42], our study differs fundamentally in that we were specifically investigating the activity of CB-839 in combination with PIs. We speculate that CB-839 induces a specific set of molecular effects that diverges from those induced by withdrawing all or part of glutamine from cells. In support of this, previous studies by others have also reported differences between CB-839 treatment and glutamine withdrawal in the context of synergy with the Bcl-2 inhibitor venetoclax [43]. Inhibition of GLS blocks the metabolism of glutamine to glutamate, and glutamate is then used in the mitochondrial TCA cycle, for the synthesis of glutathione, and the synthesis of other amino acids. However, the cell is also able to utilize glutamine directly without first converting it to glutamate (i.e. GLS independent glutamine utilization). For example, glutamine is used directly as a building block in the *de novo* synthesis of purine and pyrimidine nucleotides [44], and in the hexosamine biosynthesis pathway that produces uridine diphosphate *N*-acetylglucosamine (UDP-GlcNAc) for support of protein glycosylation, folding and trafficking [45]. We therefore conclude that the PI sensitizing activity of CB-839 derives from specific molecular events associated with inhibiting the metabolism of glutamine to glutamate while conserving the GLS independent cellular utilization of glutamine. Future studies identifying metabolite profiles specific to CB-839 treatment but distinct from glutamine deprivation may yield a more complete mechanistic understanding of the anti-MM efficacy of CB-839/PI combination therapy, and may produce metabolite biomarkers for predicting a positive clinical response to GLS inhibitors in MM.

## MATERIALS AND METHODS

### Cell lines and reagents

PI resistant MM.1S BzR and U266 BzR were a generous gift from Dr. Brian Van Ness of the University of Minnesota. These cells were selected for bortezomib (Btz) resistance as described previously and show cross-resistance to other PIs (Supplementary Figure 1) [46]. RPMI-8226 BzR cells were selected for Btz resistance by exposure to progressively higher concentrations of Btz for > 6 months. Btz concentrations were started at 1 nM and escalated by increments of 1 nM if and when culture densities doubled in cell number. RPMI-8226 BzR show roughly 6-fold resistance to the cytotoxic effects of Btz and other PIs (Supplementary Figure 1). U266, OPM2, RPMI-8226, MM.1S, ANBL6 KMS12-BM, NCI-H929, KMS12-PE, and KMS11 cells were purchased from American Type Culture Collection (ATCC) and maintained in the recommended growth media at 37°C and 5% CO_2_. MM cells were cultured in RPMI-1640 (HyClone SH30255.01) supplemented with 15% FBS, 100 U/mL penicillin, 100 μg streptomycin, 0.25 μg amphotericin, 2 mM glutamine, and 1 mM sodium pyruvate. RPMI-8226 and OPM2 cells were cultured in RPMI-1640 (HyClone SH30027.01) supplemented with 10% FBS, 100 U/mL penicillin, 100 μg streptomycin, and 0.25 μg amphotericin. Non-myeloma cells were grown in DMEM (ATCC #61978590) and supplemented with 10% FBS, 100 U/mL penicillin, 100 μg streptomycin, and 0.25 μg amphotericin. Cells that were deprived glutamine were cultured in glucose- and glutamine-free RPMI-1640 (Biological Industries #1533342) and supplemented with 15% FBS, 2 g/L glucose, 100 U/mL penicillin, 100 μg streptomycin, and 0.25 μg amphotericin. CB-839 was kindly provided by Dr. Susan Demo of Calithera Biosciences Inc. Unless stated otherwise, CB-839 was used at a concentration of 5 μM, which is within range of what is achievable in the blood plasma of patients [47].

### PSMB5 gene sequencing

We sequenced a region of Exon 2 of the *PSMB5* gene, which encodes the highly conserved substrate/inhibitor binding domain for proteasome inhibitors within the beta-5 protein. We sequenced genomic DNA from MM.1S BzR, U266 BzR, and RPMI-8226 BzR cells, and their parental counterparts, looking for the G322A (Ala49Thr), C323T (Ala49Val), and C326T conjoined mutation (Ala49Thr and Ala50Val) that have previously been implicated in the acquisition of bortezomib resistance [4, 27]. Primer sequences from these previous studies were used for sequencing. DNA was PCR-amplified, and bi-directional Sanger sequencing was performed at the University of Minnesota Genomics Center (UMGC). Multiple sequence alignment was performed against the human reference sequence GRCh37/hg19 using Sequencher v5.4.6 software.

### Measurement of mitochondrial respiration rates

Oxygen Consumption Rate (OCR) measurements were performed using a Seahorse Bioscience XF-96 instrument as previously described [48–50]. The XF96 protocol consisted of basal OCR (1 measurement/1.5 min), injection of oligomycin (0.01–1.0 mM), injection of FCCP (0.5 mM) with four measurements of uncoupled OCR (1 measurement/1.5 min), and a final injection of rotenone (1 mM) and antimycin A (1 mM). MM cells were plated at a density of 0.3 × 10^6^ cells per well in 100 μL XF running buffer into a 96-well plate. Cells treated with CB-839 were pretreated with 5 μM drug for 16 hours. Cells deprived glutamine were plated in glutamine-free XF running buffer.

### Western blot

MM cells were plated at a density of 0.8 × 10^6^ cells per 1.5 mL of medium. The cells were treated as indicated and maintained at 37°C for the indicated duration of the experiment. Cells were lysed in 1X Laemmli sample buffer. Proteins were separated by SDS-PAGE using NuPage 4–12% bis-tris gradient gels with MOPS buffer and then transferred to PVDF membranes. The following antibodies were used for Western blotting: P-AMPKα (Cell Signaling #2535P), AMPKα (Cell Signaling #583P), P-ACC (Cell Signaling #11818P), Cytochrome C (Cell Signaling #4280P), SDHα (Cell Signaling #11998P), Cox IV (Cell Signaling #4850P), GAPDH (Cell Signaling #5174P), Histone H3 (Cell Signaling #9715S), PARP (Cell Signaling #9532), Caspase-3 (Cell Signaling #9662), Caspase-8 (Cell Signaling #9746), ATF-4 (Cell Signaling #11815), CHOP (Cell Signaling #2895), GCN2 (Cell Signaling #3302S), and β-actin (Cell Signaling #3700) at a 1:1000 dilution. For experiments comparing the effects of glutamine withdrawal to CB-839 treatment, cells were plated in media with or without glutamine and treated with DMSO (vehicle control) or CB-839. Treatments were initiated at the same time in order to accurately compare the two conditions. Cells were then immediately exposed to Crflz for the indicated time points. Band densitometry was analyzed using ImageJ software.

### NAD(P)H assay

Steady state cellular NAD(P)H levels were measured in intact cells over time using a QM-4 fluorimeter (PTI, Piscatway, NJ) in kinetic mode. The reduced but not oxidized forms of these electron carriers have inherent florescence properties (Ex: 340 nm, Em: 460 nm). Fluorescence intensity data were collected every 0.1 seconds over a period of 100 seconds for PI sensitive and resistant isogenic MM cell lines, and the average ± S.E. was calculated.

### Measurement of mitochondrial biomass

Mitochondria were stained by incubating live MM.1S and MM.1S BzR cells with the MitoTracker Green FM probe (Molecular Probes, 200 nM) according to the manufacturer's instructions. Cells were washed and seeded in triplicate at varying cell densities in 200 μL of growth medium. Fluorescence was quantified using a Spectramax i3 multimode microplate reader (Molecular Devices; Ex: 490 nm, Em: 516 nm). Alternatively, cells were incubated with a dose range of the MitoTracker Green FM probe and a fixed number of cells (0.2 × 10^6^) were plated. Fluorescence intensity was measured as described above.

### Patients

Comparative proteome analysis was performed on plasma cells (PCs) obtained from pretreatment bone marrow from 24 patients (median age 67 [52–75] with relapsed refractory MM qualified for PAD (bortezomib, doxorubicin, dexamethasone) therapy [51]. Disease response to treatment was assessed by International Myeloma Working Group (IMWG) criteria [52]. Patients received a median of 6 cycles (range 2–8). Treatment was interrupted due to toxicity (6 patients, grade 3 neuropathy, grade 2 infection) and disease progression (4 patients). After PAD, 3 patients achieved a complete response (CR, 13%), 7 achieved a very good partial response (VGPR, 30%), 8 achieved a partial response (PR, 33%), and 6 had progressive disease (PD, 25%). There were no patient deaths during the course of treatment.

### MM patient plasma cell isolation

Bone marrow samples were taken after patient informed consent and were freshly purified by ficoll paque gradient separation and CD138-positive plasma cells were isolated using the EasySepÔ immunomagnetic separation platform in conjunction with the EasySepÔ Human CD138 Positive Selection Kit (Stem Cell Technology, USA) according to manufacturer's protocol. PCs were frozen as 0.5 × 10^6^ cells per pellet in liquid nitrogen and kept until proteomic analysis.

### Label-free based proteomic approach

Plasma cells were lysed in 1 M triethylammonium bicarbonate (TEAB) and 1% SDS and homogenized using Precellys 24 homogenizer (Bertin Technologies, France) in 0.5 mL tubes pre-filled with ceramic (zirconium oxide) beads (Bertin Technologies, France). For all homogenization, 20 μl of buffer was added to each 0.1 × 10^6^ cells and processed at 6,300 rpm for 30 seconds in three cycles. Material was then sonicated in three 1 minute cycles on ice and again homogenized in Precellys 24. Homogenized suspension was centrifuged at 16,000xg for 10 minutes at 4°C and supernatants were retained for analysis. Protein concentrations were determined using a BCA (Pierce) method. Ten μg of plasma cell proteins were reduced in the presence of 50 mM NH_4_HCO_3_ with 5.6 mM DTT for 5 min at 95°C. Then, the sample was alkylated with 5 mM iodoacetamide for 20 min in the dark at room temperature. The proteins were digested with 0.2 μg of sequencing-grade trypsin (Promega, Germany) overnight at 37°C. Each sample was prepared for digestion in duplicate.

### NanoLC-MS/MS analysis

For each run, 1.5 μg of the digested protein samples was injected onto an RP C18 precolumn (Thermo Fisher Scientific, USA) connected to a 75 μm i.d. x 25 cm RP C18 Acclaim PepMap column with a particle size of 2 μm and a pore size of 100 Å (Thermo Fisher Scientific, USA) using a Dionex UltiMate 3000 RSLCnano System (Thermo Fisher Scientific, USA). Every sample was injected in duplicate at random. Every 13 sample injections, the system was calibrated using Pierce LTQ ESI Positive Ion Calibration Solution (Thermo Fisher Scientific, USA). Then, 13 freshly digested samples were injected without any break. The following LC buffers were used: buffer A (0.1% (v/v) formic acid in Milli-Q water) and buffer B (0.1% formic acid in 90% acetonitrile). The peptides were eluted from the column with a constant flow rate of 300 nL min^−1^ with a linear gradient of buffer B from 5% to 65% over 208 min. At 208 min, the gradient increased to 90% B and was held there for 10 min. Between 218 and 230 min, the gradient returned to 5% to re-equilibrate the column for the next injection. The peptides eluted from the column were analyzed in the data-dependent MS/MS mode on a Q-Exactive Orbitrap mass spectrometer (Thermo Fisher Scientific, USA). The instrument settings were as follows: the resolution was set to 70,000 for MS scans, and 17,500 for the MS/MS scans to increase the acquisition rate. The MS scan range was from 350 to 2,000 m/z. The MS AGC target was set to 1e6 counts, whereas the MS/MS AGC target was set to 5e4. Dynamic exclusion was set with a duration of 20 s. The isolation window was set to 2 m/z. Normalized collision energy was set to 28.

### Qualitative analysis of label-free proteomic data

After each LC-MS/MS run, the raw files were qualitatively analyzed by Proteome Discoverer (PD), version 1.4.14 (Thermo Fisher Scientific, USA). To evaluate the quality of the performed runs, the number of peptide spectrum matches (PSMs) and the number of identified proteins were calculated. The LC-MS/MS runs with the number of PSMs below 125,000 and the number of identified proteins below 1,000 (with 1% FDR) were excluded from further analysis. The identification of proteins by PD was performed using the SEQUEST engine against the UniProt Complete Proteome Set of Humans (123,619 sequences) using the following parameters: a tolerance level of 10 ppm for MS and 0.05 Da for MS/MS. Trypsin was used as the digesting enzyme, and two missed cleavages were allowed. The carbamidomethylation of cysteines was set as a fixed modification, and the oxidation of methionines was allowed as a variable modification.

### Quantitative analysis of label-free proteomic data

The raw files positively evaluated by PD were quantitatively analyzed by MaxQuant (Cox and Mann 2008; Cox et al. 2011), version 1.5.1.2. The database search engine Andromeda was used to search the MS/MS spectra against the UniProt database, with the same parameters as for PD at ≤ 1% FDR. The analysis of the samples was based on the label-free quantification (LFQ) intensities. The data were evaluated, and the statistics were calculated using Perseus software (version 1.4.1.3, Max Planck Institute of Biochemistry, Martinsried). The MQ data were filtered for reverse identifications (false positives), contaminants, and proteins “only identified by site.” The mean LFQ intensities as well as the standard deviation of this value were calculated for both experimental groups. The fold changes in the level of the proteins were assessed by comparing the mean LFQ intensities among both experimental groups. A protein was considered to be differentially expressed if the difference was statistically significant (*p* < 0.05), the fold change of minimum was +/− 1.5, it was identified with a minimum of 2 peptides with > 99% confidence.

### Cell viability and apoptosis assays

Cell viability and apoptosis were measured in 96 and 384-well cell culture plates using the Cell Titer-Glo Luminescent Cell Viability Assay (Promega) and the Caspase-Glo 3/7 Assay (Promega), respectively, according to the manufacturers protocol. Cells were treated with a maximum of 0.1% DMSO as a vehicle control or the indicated drug for the indicated time at 37°C. Luminescence was recorded on the SpectraMax L Microplate Reader (Molecular Devices) at 470 nm with a 1-second integration time. Dose response curves were generated to calculate the half maximal effective concentration (EC_50_) in GraphPad Prism version 6.07. For synergy studies, a fixed concentration of CB-839 was used in combination with an 8-dose range of PI for 24 hours. When studies were conducted to assess the effects of CB-839 treatment alone on MM cell viability or under glutamine-deprived conditions without the presence of Crflz, longer treatment times of 72 hours were used, unless otherwise indicated. In each case, data were normalized to factor out any effect of CB-839 by itself, and establish a true and relative EC_50_ for the PI in the presence or absence of CB-839. Therefore, any leftward shift of the PI dose response curve is indicative of a super-additive and synergistic drug interaction with CB-839.

## SUPPLEMENTARY MATERIALS FIGURES AND TABLES


