# Supplementary material for: Glutaminase inhibitor CB-839 synergizes with carfilzomib in resistant multiple myeloma cells
Source: Oncotarget. 2017 Mar 16;8(22):35863–76. doi: 10.18632/oncotarget.16262 (PMC5482623; doi:10.18632/oncotarget.16262)
Supplement: Supplementary file 1 [file oncotarget-08-35863-s001.pdf]

Glutaminase inhibitor CB-839 synergizes with carfilzomib in resistant multiple myeloma cells

Supplementary Materials

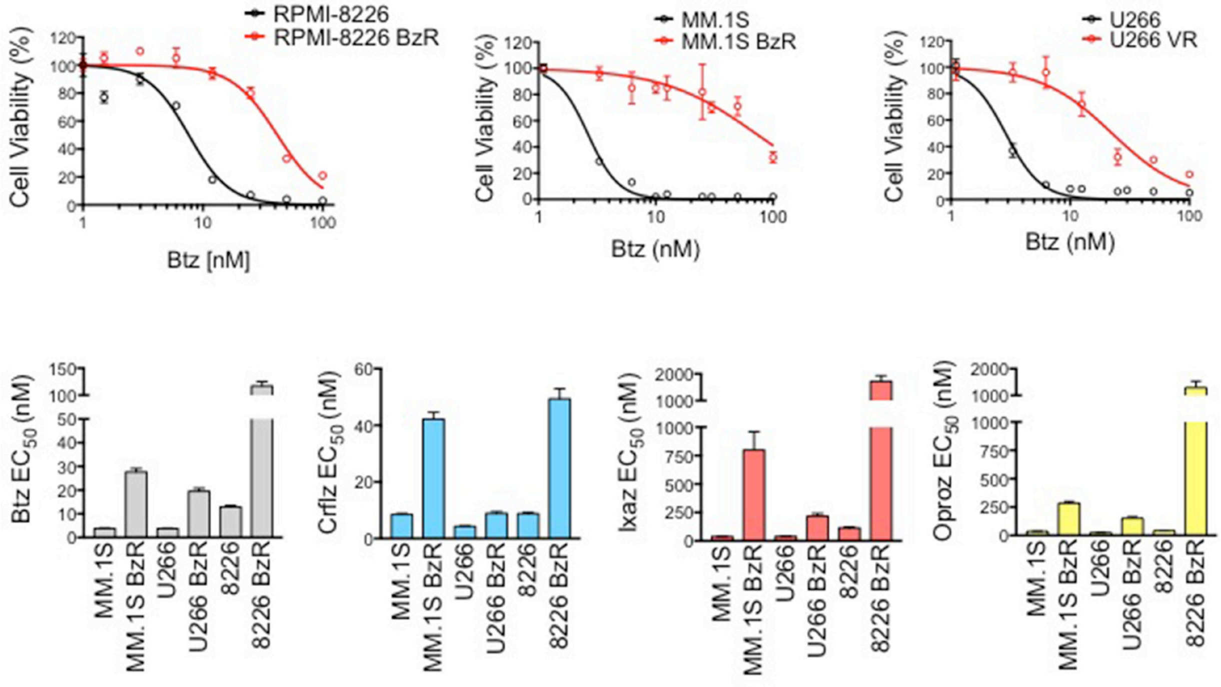

Supplementary Figure 1: Characterization of PI resistant MM cell models. RPMI-8226 BzR, MM.1S BzR, and U266 BzR cells were generated by exposing PI sensitive RPMI-8226, MM.1S, and U266 cells to progressively higher concentrations of bortezomib (Btz) for > 6 months. All cell lines were treated with a dose range of Btz, carfilzomib (Crflz), ixazomib (Ixa), or oprozomib (Oproz) for 24 hours. Cell viability data are shown with representative dose-range curves of Btz (top), and EC50 values for all PIs quantified (bottom).

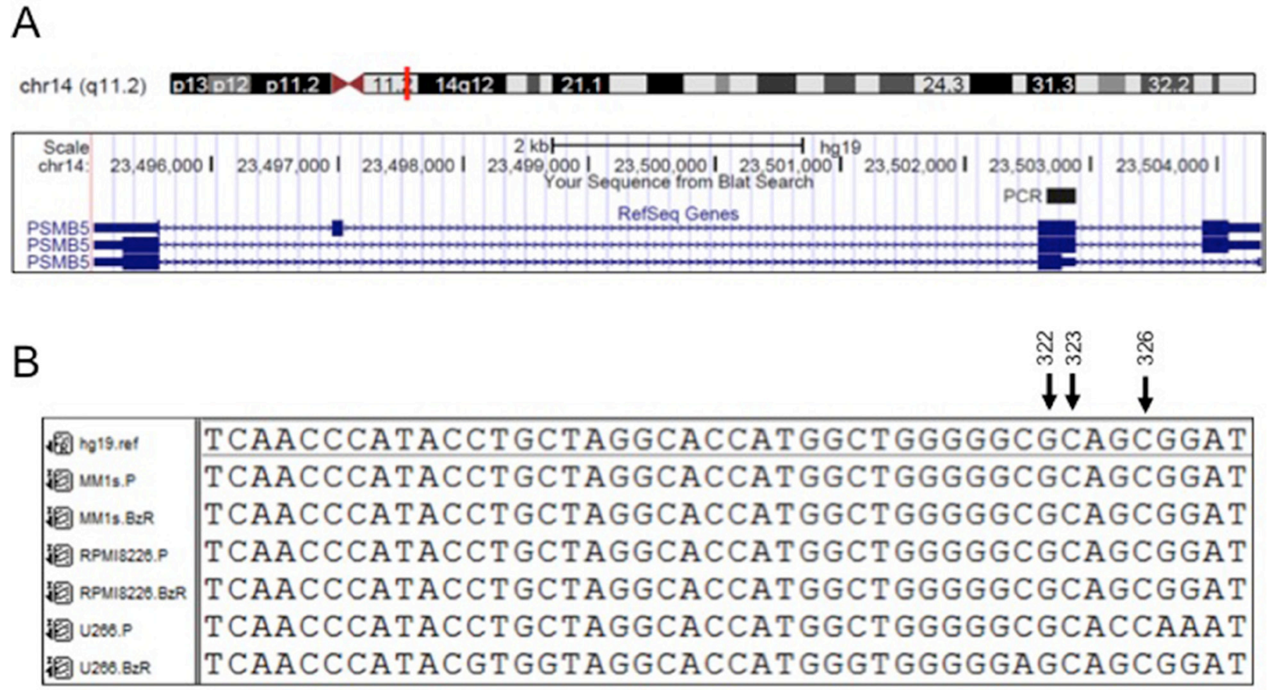

Supplementary Figure 2: PI resistant MM cells lack PSMB5 gene mutations. Genomic DNA was extracted from Paired PI sensitive and resistant (MM.1S/MM.1S BzR, RPMI-8226/RPMI-8226 BzR, and U266/U266 BzR). (**A**) A region of *PSMB5* that encodes the PI binding region in the beta-5 proteasomal subunit protein was sequenced. (**B**) Sequences were analyzed for the G322A (Ala49Thr), C323T (Ala49Val), and C326T conjoined mutation (Ala49Thr and Ala50Val) mutations that have been reported by others in cell models of PI resistance.

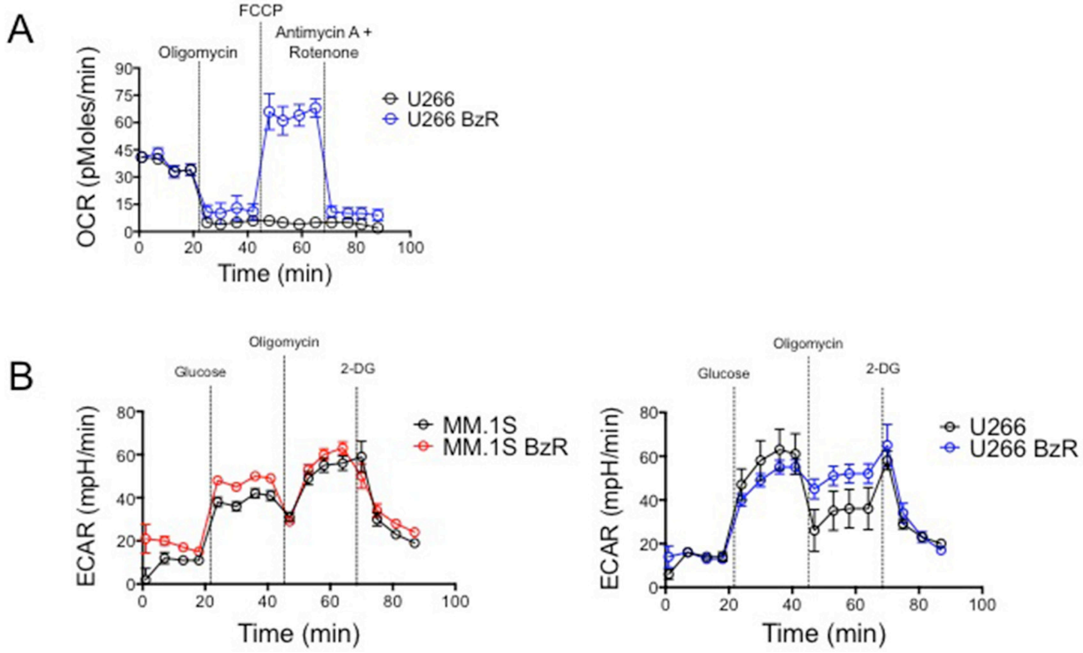

Supplementary Figure 3: Oxygen consumption and glycolysis rates in PI sensitive versus resistant MM cells. (**A**) Oxygen consumption rates (OCR), a measure of cellular glycolysis, are shown for PI sensitive U266 and PI resistant U266 BzR cells. (**B**) Extracellular acidification rates (ECAR), a measure of cellular glycolysis, are shown for PI sensitive (MM.1S and U266) and PI resistant (MM.1S BzR and U266 BzR) cells.

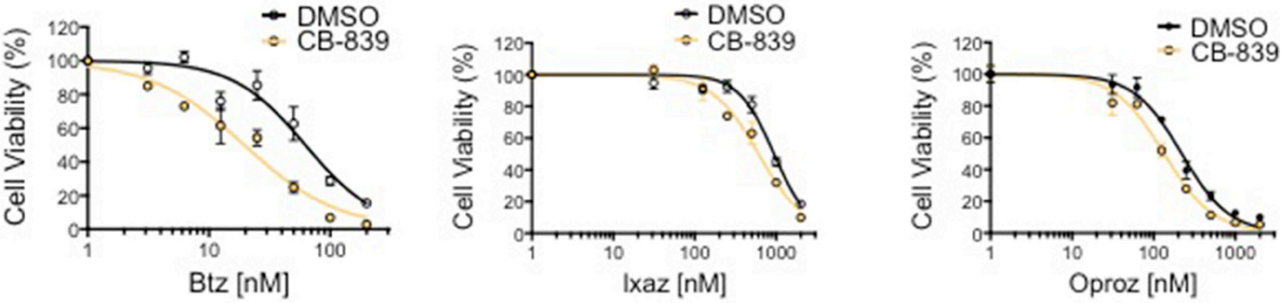

Supplementary Figure 4: Glutaminase-1 inhibitor, CB-839, enhances PI sensitivity in resistant MM cells. MM.1S BzR cells were exposed to dose ranges of bortezomib (Btz), ixazomib (Ixaz), and oprozomib (Oproz) for 24 hours in the presence or absence of 5 µM CB-839. Cell viability data are shown.

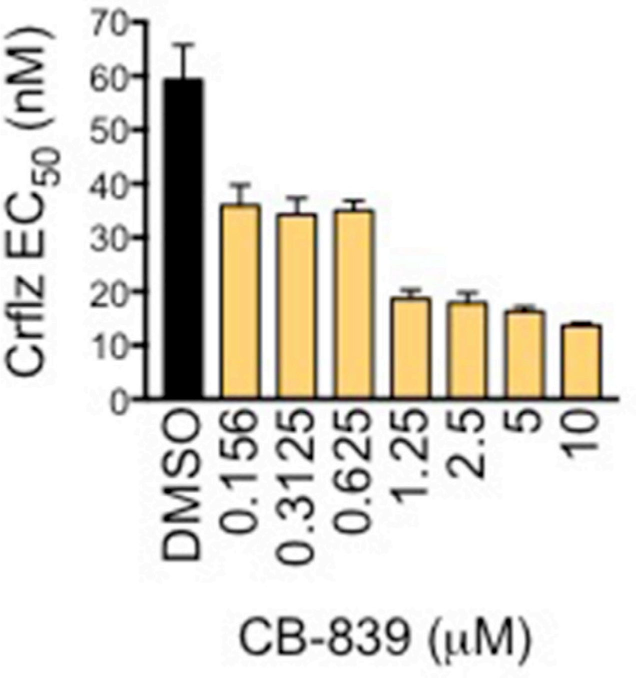

Supplementary Figure 5: CB-839 synergizes with Carfilzomib in resistant MM cells. MM.1S BzR cells were exposed to dose ranges of both CB-839 (0-10 µM) and carfilzomib (Crflz, 0-200 nM) for 24 hours. The half maximal concentration (EC50) of Crflz at each dose of CB-839 is shown here ($N = 3$).

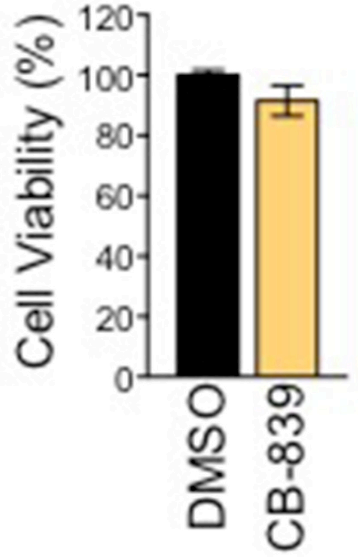

Supplementary Figure 6: Cell viability of MM.1S BzR cells. MM.1S BzR cells were exposed to DMSO or CB-839 (5 μM) for 24 hours. Data are represented as percent cell viability mean + SEM from 4 independent experiments (*N* = 12). There was no statistically significant difference between groups using a student's t-test (*P* = 0.144).

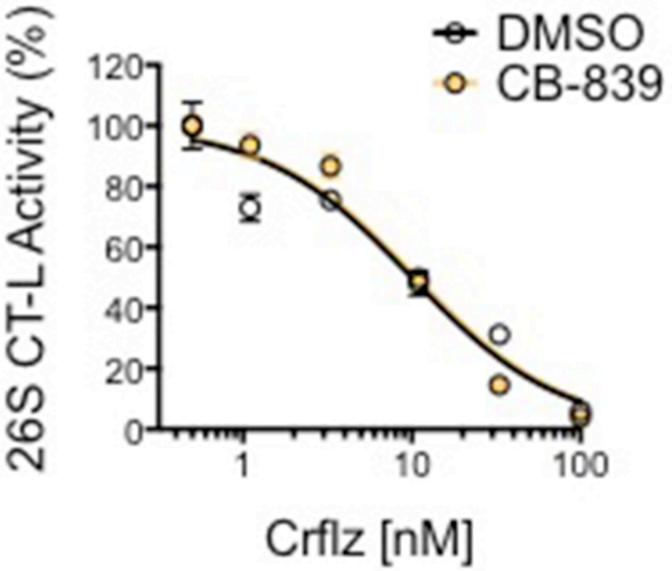

Supplementary Figure 7: Measurement of chymotrypsin-like (CT-L) activity of the 26S proteasome. CT-L activity was measured in MM.1S BzR cells treated with a dose range of carfilzomib (Crflz, 0-200 nM) in addition to CB-839 (5 μM) or DMSO as a vehicle control for 24 hours. CT-L activity of the 26S proteasome is shown.

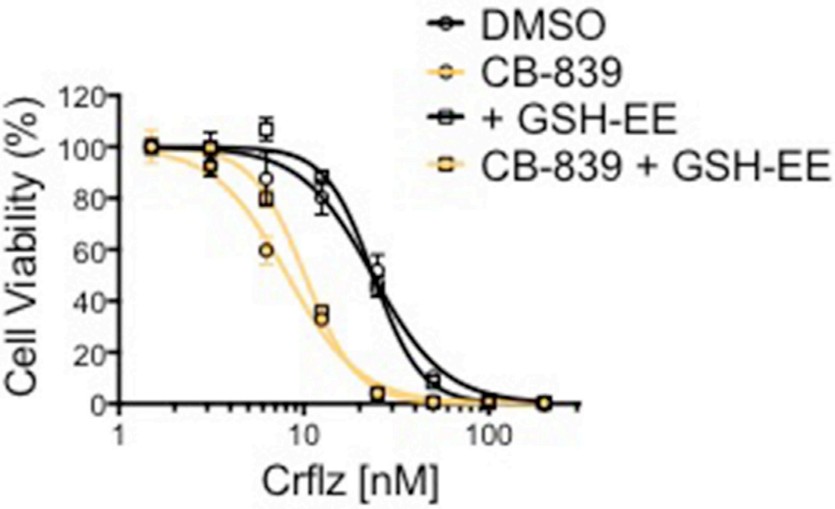

Supplementary Figure 8: Glutathione ethyl esterase does not augment cell viability after CB-839 treatment. MM.1S BzR cells were treated with a dose range of carfilzomib (Crflz, 0-200 nM) in addition to CB-839 (5 μM) and/or glutathione ethyl esterase (GSH-EE, 2 mM, Sigma Aldrich) for 24 hours. Cell viability data is shown.

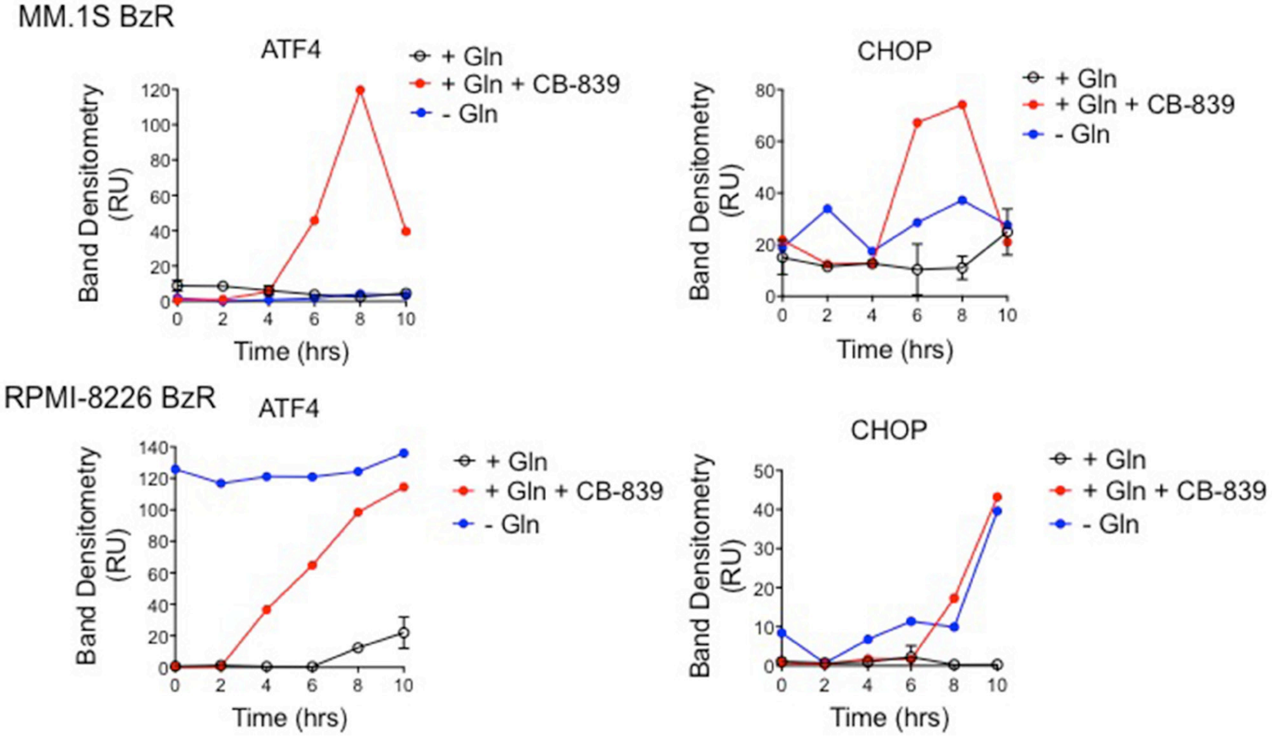

Supplementary Figure 9: Densotometric band quantification of western blots from Figure 5C. MM.1S BzR cells (top) and RPMI-8226 BzR cells (bottom) were treated for the indicated time points with Crflz (20 nM) alone or in combination with CB-839 (5 μM). For comparison, similar experiments were conducted in media containing or lacking glutamine (4.0 mM). The band densitometry is shown here (+Gln : Crflz treatment alone; CB-839 + Gln: Crflz and CB-839 in the presence of Gln; -Gln: Crflz treatment in the presence of no Gln).

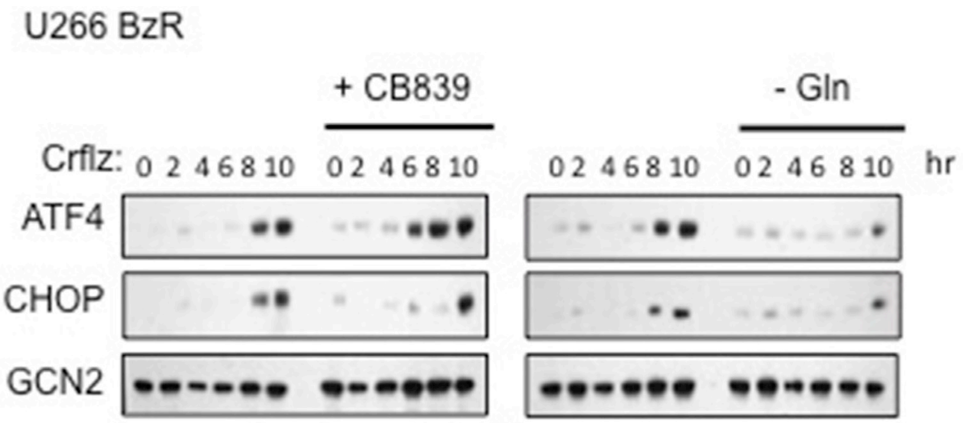

Supplementary Figure 10: Characterization of ER stress markers in PI resistant U266 BzR cells. U266 BzR cells were treated for the indicated time points with Crflz (20 nM) alone or in combination with CB-839 (5 µM). For comparison, similar experiments were conducted in media containing or lacking glutamine (4.0 mM). To accurately compare the 2 different conditions, glutamine-free incubation and addition of CB-839 coincided with the beginning of Crflz treatment. Western blots are shown. GCN2 was used as a loading control.

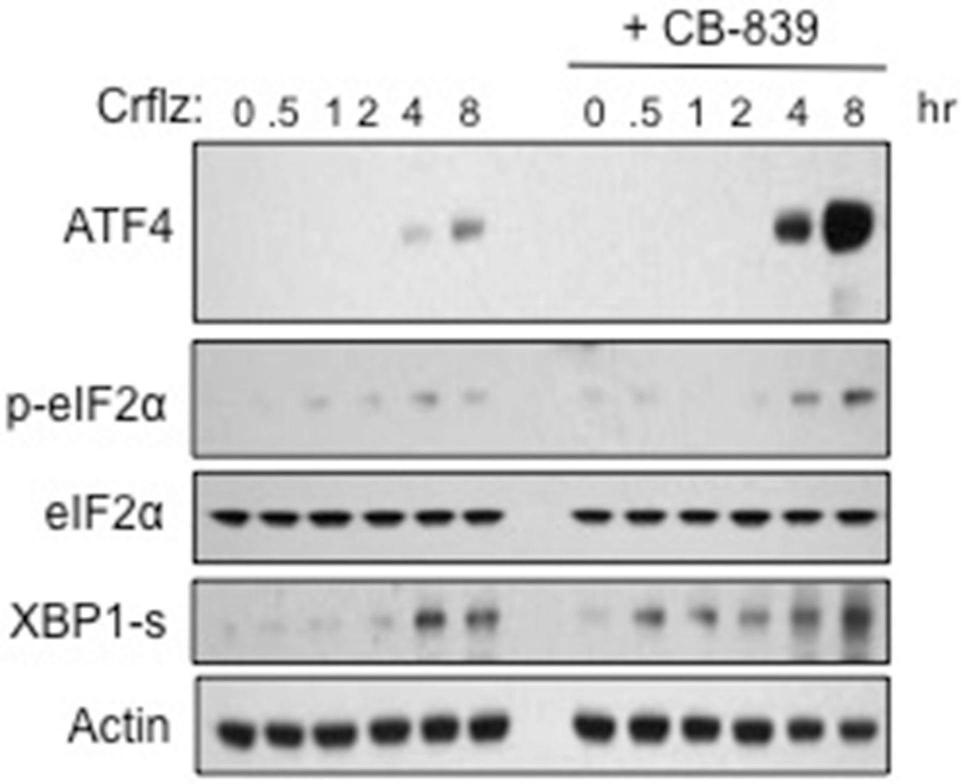

Supplementary Figure 11: Characterization of ER stress pathway activated in PI resistant RPMI-8226 BzR cells. RPMI-8226 BzR cells were treated for the indicated time points with Crflz (20 nM) alone or in combination with CB-839 (5 µM). Western blots are shown. Actin was used as a loading control.

Supplementary Table 1A: Proteins down-regulated in non-responder patient plasma cells

Protein Name	Gene	Non-responder/Responder	*P*
Calmodulin	CALM1	0.66	1.97E-02
High mobility group protein B1	HMGB1	0.65	4.07E-03
Lamin-B1	LMNB1	0.64	4.45E-02
Cathepsin D; Cathepsin D light chain; Cathepsin D heavy chain	CTSD	0.64	2.47E-02
Lysosome-associated membrane glycoprotein 2	LAMP2	0.62	9.27E-03
Ras-related protein Rab-14	RAB14	0.58	2.80E-04
Ras-related protein Rap-1b	RAP1B	0.57	5.25E-03
DnaJ homolog subfamily C member 1	DNAJC1	0.56	6.03E-04
Lymphocyte-specific protein 1	LSP1	0.56	5.65E-04
Serine-tRNA ligase, cytoplasmic	SARS	0.55	1.37E-03
Glutathione S-transferase P	GSTP1	0.53	1.98E-03
Catalase	CAT	0.52	8.73E-03
Vimentin	VIM	0.52	2.68E-03
Rho GDP-dissociation inhibitor 2	ARH GDIB	0.52	2.22E-02
High mobility group protein B2	HMGB2	0.50	9.17E-03
Protein LYRIC	MTDH	0.48	1.58E-03
Myosin regulatory light chain 12A	MYL12A	0.47	1.63E-02
Translocating chain-associated membrane protein	TRAM1	0.47	4.50E-04
Phosphoserine aminotransferase	PSAT1	0.46	4.08E-03
Ferritin light chain; Ferritin	FTL	0.40	4.28E-02
Cathepsin B	CTSB	0.38	4.25E-02
Galectin-1	LGALS1	0.37	3.12E-03
Prelamin-A/C	LMNA	0.37	2.85E-03
Neutrophil elactase	ELANE	0.36	1.74E-02
Prosaposin	PSAP	0.36	1.98E-03
Myosin-9	MYH9	0.36	3.33E-03
Tyrosine-protein kinase receptor	TPM3	0.35	4.93E-03
Ferritin	FTH1	0.34	1.24E-02
Filamin-A	FLNA	0.28	4.25E-02
Neutrophil defensin 3	DEFA3	0.28	2.00E-03
Plastin-2	LCP1	0.28	2.06E-03
Plectin	PLEC	0.27	1.57E-02
Protein S100-A8; Protein S100-A8, N-terminally processed	S100A8	0.25	1.90E-02
Myeloperoxidase	MPO	0.22	5.43E-03
Protein S100-A9	S100A9	0.21	1.01E-02
Annexin	ANXA1	0.21	5.98E-04
Lactotransferrin	LTF	0.14	1.64E-03

Supplementary Table 1B: Proteins enriched in non-responder patient plasma cells

Protein Name	Gene	Non-responder/Responder	*P*
Cytochrome b-c1 complex subunit 1, mitochondrial	UQCRC1	4.01	1.17E-02
Proteasome activator complex subunit 2	PSME2	3.75	4.03E-05
Proteasome activator complex subunit 1	PSME1	3.06	1.47E-04
Thioredoxin	TXN	2.91	5.92E-03
Lactoylglutathione lyase	GLO1	2.74	4.27E-02
Macrophage migration inhibitory factor	MIF	2.45	2.80E-03
Very long-chain specific acyl-CoA dehydrogenase, mitochondrial	ACADVL	2.42	8.48E-04
ATP synthase subunit O, mitochondrial	ATP5O	2.24	5.11E-03
Cytochrome b-c1 complex subunit 2, mitochondrial	UQCRC2	2.17	4.97E-02
HLA class II histocompatibility antigen gamma chain; Tyrosine-protein kinase receptor	CD74	2.12	6.45E-04
Stress-70 protein, mitochondrial	HEL-S-124m	2.12	1.22E-04
Citrate synthase; Citrate synthase, mitochondrial	CS	2.09	5.22E-03
Voltage-dependent anion-selective channel protein 1	VDAC1	2.05	4.74E-02
Von Hippel-Lindau disease tumor suppressor	VHL	1.99	2.42E-02
Stress-induced-phosphoprotein 1	HEL-S-94n	1.97	3.51E-02
Glutathione S-transferase kappa 1	LOC51064	1.95	6.61E-03
Cytosolic non-specific dipeptidase	HEL-S-13	1.95	4.93E-02
Elongation factor Tu, mitochondrial	TUFM	1.93	4.32E-03
Heterogeneous nuclear ribonucleoprotein M	HNRNPM	1.85	1.19E-02
Matrin-3	MATR3a	1.84	2.61E-03
ATP synthase subunit alpha; ATP synthase subunit alpha, mitochondrial	ATP5A1	1.83	5.93E-03
Cytochrome c	CYCS	1.81	2.54E-02
Thioredoxin-dependent peroxide reductase, mitochondrial	PRDX3	1.74	2.87E-02
Peroxiredoxin-5, mitochondrial	PRDX5	1.71	1.43E-03
Protein disulfide-isomerase A6	PDIA6	1.69	1.31E-02
Adenosylhomocysteinase	AHCY	1.69	1.54E-02
Glutathione S-transferase omega-1	GSTO1	1.66	1.08E-02
ATP synthase subunit beta; ATP synthase subunit beta, mitochondrial	ATP5B	1.65	8.25E-03
40S ribosomal protein S10; Putative 40S ribosomal protein S10-like	RPS10	1.64	4.98E-02
Peroxiredoxin-6	PRDX6	1.64	4.69E-02
Thioredoxin domain-containing protein 5	TXNDC5	1.63	2.20E-02
14-3-3 protein epsilon	HEL2	1.62	1.41E-02
Peroxiredoxin-2	PRDX2	1.58	4.33E-02
X-ray repair cross-complementing protein 5	XRCC5	1.55	7.80E-03
Neutral alpha-glucosidase AB	GANAB	1.53	1.76E-02
X-ray cross-complementing protein 6	XRCC6	1.51	4.50E-02

Supplementary Table 2: EC_{50} values of MM cells and normal human primary cells treated with Crflz or CB-839 + Crflz

Cell Line	Crflz EC50 (nM)	Crflz + CB-839 EC50 (µM)	Fold Change
U266	26.2	7.7	3.5
OPM2	39.5	12.6	3.2
RPMI8226	15.0	5.8	2.9
MM.1S BzR	37.4	14.5	2.6
MM.1S	5.6	2.3	2.5
595sp	71.1	31.9	2.2
ANBL6	16.6	8.3	2.0
ANBL6-Vec	22.5	14.8	1.52
RPMI8226.BzR	51.1	39.2	1.32
KMS12 BM	52.6	42.1	1.32
H929	3.2	2.6	1.25
U266 BzR	17.8	16.1	1.13
KMS12 PE	13.0	11.9	1.11
KMS11	20.7	21.2	1.0031
595sp BzR	8859.0	11522.0	–1.21
ANBL6-NRAS	10.7	18.7	–1.43
PBMC	35.5	12.3	2.89
WBC	33.1	21.8	1.52
HEK	467.9	570.5	–1.18
MEF	587.2	1231.0	–1.32
Wi38	79.2	156.8	–1.47
NIH/3T3	211.2	5183.0	–1.79

Cells were treated for 24 hours with a dose range of Crflz and the addition of DMSO or CB-839 (5 µM). The EC_{50} value was calculated and is reported here along with fold change ($N = 3$).
